# Self-Encapsulation of Biomacromolecule Drugs in Porous Microscaffolds with Aqueous Two-Phase Systems

**DOI:** 10.3390/pharmaceutics13030426

**Published:** 2021-03-22

**Authors:** Jian Kang, Yunpeng Cai, Ziwei Wu, Siyi Wang, Wei-En Yuan

**Affiliations:** Engineering Research Center of Cell & Therapeutic Antibody, Ministry of Education, School of Pharmacy, Shanghai Jiao Tong University, Shanghai 200240, China; jkang@sjtu.edu.cn (J.K.); cai_yunpeng@wuxiapptec.com (Y.C.); vero_shero@sjtu.edu.cn (Z.W.); wangnima@sjtu.edu.cn (S.W.)

**Keywords:** self-healing, porous microscaffolds, aqueous two-phase system, biomacromolecule drugs, closed pores

## Abstract

At present, the most commonly used methods of microencapsulation of protein drugs such as spray drying, multiple emulsification, and phase separation, can easily cause the problem of protein instability, which leads to low bioavailability and uncontrolled release of protein drugs. Herein, a novel method to encapsulate protein drugs into porous microscaffolds effectively and stably was described. Ammonium hydrogen carbonate (NH_4_HCO_3_) was employed to prepare porous microscaffolds. α-Amylase was encapsulated into the porous microscaffolds without denaturing conditions by an aqueous two-phase system (PEG/Sulfate). The pores were closed by heating above the glass transition temperature to achieve a sustained release of microscaffolds. The pore-closed microscaffolds were characterized and released in vitro. The integrity and activity of protein drugs were investigated to verify that this method was friendly to protein drugs. Results showed that the pores were successfully closed and a high loading amount of 9.67 ± 6.28% (*w/w*) was achieved. The pore-closed microscaffolds released more than two weeks without initial burst, and a high relative activity (92% compared with native one) of protein demonstrated the feasibility of this method for protein drug encapsulation and delivery.

## 1. Introduction

Biodegradable polymeric microparticles/microscaffolds mainly based on poly (D, L-lactic-co-glycolic acid) (PLGA) have been extensively studied as an injectable sustained release depot for protein delivery over the decades [1]. However, the most commonly used methods of microencapsulation of protein drugs are spray drying, multiple emulsion, and phase separation, which can introduce organic solvent–water interfaces, shear induced stress during emulsification, resulting in structure unfold, aggregation, and denaturation of proteins [2,3,4]. Protein instability leads to low bioavailability and uncontrolled release of protein drugs loaded microparticles/microscaffolds, which makes protein stability an urgent problem.

To address the stability issue, some researchers have turned to porous microparticles/microscaffolds, which have a high capacity for protein loading due to its porous structure [5]. Whitely M. et al. found that high protein loading efficiencies were achieved in porous microspheres. Although there is a short period of explosive release, the protein sustained released with minimal burst release [6]. Protein is encapsulated into porous microparticles/microscaffolds to avoid the problems caused by traditional preparation methods. After the protein is loaded, the pores of the particles are closed to form a sustained release depot.

Next, the question turned into how to encapsulate the protein and close the pores. To close the pores, heating to glass transition temperature (Tg) or above can trigger a “self-healing” process based on the mobility of the polymer chains. The mechanism includes polymer-chain inter-diffusion driven by minimization of the energetically unfavorable interfacial area and/or transfer of potential energy stored in the defect [7]. Hence, the loading efficiency is usually low without the driving force, which drives the protein into the pores. Desai and Schwendeman used Al(OH)_3_ as an antigenic adjuvant, increased the loading amount to about 1.3% (*w/w*), and heated it in water to close the pores [8]. However, the employment of an adjuvant cannot be regarded as a potent strategy to increase the loading amount in porous microparticles. Kim et al. used ethanol vapor in a fluidized bed to close the pores and achieved a high loading of 3.1 ± 0.1% (*w/w*) of human growth hormone [9]. However, the exposure to organic solvent remains a question.

Herein, we describe a loading strategy with an aqueous two-phase system (ATPS), greatly increasing the loading amount and avoiding any factor to denature the proteins. ATPS is available in several combinations such as two polymers, one polymer, and one salt or two salts, which are low cost and high efficiency and have been used for decades in biotechnology applications as non-denaturating and initial separation media [10,11,12,13,14]. One of the most well-known ATPS forming combinations is poly(ethylene glycol) (PEG) and salt (such as phosphate, sulfate, and citrate), since the salt will capture a large amount of the water present. It is well understood that hydrophilic macromolecules, like proteins, generally cannot be distributed or diffused in hydrophobic polymer phases [15]. In this aqueous two-phase system, proteins will partition to the top, less polar, and more hydrophilic phase, usually PEG [16,17]. Thus, proteins can be encapsulated into porous microparticles/microscaffolds if it has a high partition coefficient in a phase that can be fixed in microparticles. Additionally, the distribution of biological macromolecules in ATPS is influenced by various factors such as their charge, molecular weight, concentration, and ionic composition of the medium [18,19].

## 2. Materials and Methods

### 2.1. Materials

PLGA 2A (lactide/glycolide = 50/50, MW: 12,000) was purchased from Surmodic Pharmaceuticals, Inc., Birmingham, AL, USA. Bovine serum albumin (BSA) (MW: 66,446 KDa), α-amylase (MW: 97,000), dinitrosalicylic acid and PEG4000 were purchased from Sigma-Aldrich (St. Louis, MO, USA). Ammonium hydrogen carbonate (NH_4_HCO_3_), ammonium sulfate, trehalose, phenol, and other reagents were purchased from Sinopharm Chemical Reagent Co. Ltd. (Shanghai, China), analytically pure.

### 2.2. Methods

#### 2.2.1. Partition of Protein Drugs in the Aqueous Two-Phase System (ATPS)

An aqueous two-phase system was prepared based on ammonium sulfate (20% *w/w*) and PEG4000 (10% *w/w*), with sodium chloride to adjust the partition. The protein solution with the concentration of 1 mg/mL was added, and the homogeneous protein solution was obtained by vortexing. The system was divided into two layers after standing for 1 h, and samples from the upper and lower phase were assayed for protein concentration. Two systems and two proteins were investigated, along with the influence of sodium chloride concentration. One system was PEG/phosphate, using potassium dihydrogen phosphate and sodium hydroxide to adjust the pH to 7.0. The other was the PEG/sulfate system, using ammonium sulfate and PEG. BSA and α-amylase were chosen as the model proteins.

The protein concentration was determined by the bicinchoninic acid (BCA) method and standard curve was used for each protein [20]. After mixing with the working solution and incubating at 37 °C for 2 h, the absorbance was measured at a wavelength of 562 nm. The detection range of protein concentration was 0.5–200 μg/mL. Ammonium sulfate and potassium phosphate could interfere with the binding of the dye Coomassie Blue G-250, resulting in a lower absorbance. In contrast, PEG elevated the absorbance slightly. Thus, the standard curve should add the same amount of PEG and salt to minimize the error (PEG: 0–200 μg/mL; salt: 40 μg/mL).

#### 2.2.2. Preparation of Porous Microscaffolds

Porous microparticles/microscaffolds were prepared by solvent evaporation degradation of sodium ammonium hydroxide [21]. PLGA was dissolved in dichloromethane to form a 15% (*w/w*) solution and 10% (*w/w*) PEG4000 was added. NH_4_HCO_3_ was dissolved in purified water, and mixed with the PLGA solution at a volume ratio of 1:5. This primary emulsion was immediately homogenized at the speed of 10,000 rpm for 1 min, and quickly transferred into 20 mL 1% PVA and 10% PEG solution to form microparticles by stirring at a rate of 300 rpm for 1 min. Then, the suspension with microscaffolds was transferred into a bulky water phase with 10% PEG. After gently stirring for 2 h, microscaffolds were collected by centrifugation and lyophilized in a freeze drier.

#### 2.2.3. Characterization of Porous Microscaffolds

The surface morphology of porous microscaffolds was observed by a scanning electronic microscope (SEM, Philips 535 M). Samples were coated with gold. Photographs of porous microscaffolds and pore closed microscaffolds were taken.

The particle size of microscaffolds was measured by a particle size analyzer (Malvern Mastersizer 2000, Malvern, UK) using a refractive index of 1.4, an absorption rate of 0.001, and water as the medium.

The glass transition temperature of porous microscaffolds was measured by a differential scanning calorimeter (Mettler Toledo^®^ DSC 1, Greifensee, Switzerland). Microscaffold samples were sealed in aluminum hermetic pans and thermograms were determined by heating from 20 °C to 100 °C at a rate of 5 °C/min under a nitrogen atmosphere.

#### 2.2.4. Protein Drug Loading and Pore-Closing Process

To encapsulate the protein drug into blank porous microscaffolds, 15 mg of microscaffolds were accurately weighed and dispersed in a solution containing PEG/sulfate system and protein drug (1 mg/mL). The suspension was incubated for 24 h in a shaker at 25 °C for protein drug loading, and another 24 h at 42 °C for pore closing [8]. Trehalose (1% *w/v*) was added to stabilize the protein drug. After incubation, microscaffolds were collected, washed three times with distilled water and lyophilized for further investigation.

#### 2.2.5. Porosity of Microscaffolds

The porosity of the samples was measured by thee nitrogen adsorption-desorption test at −196 °C using an AUTOSORB-IQ3 (Shanghai, China) specific surface area and porosity analyzer. First, the prepared blank microscaffolds and pore closed microscaffolds were degassing in a vacuum at 25 °C for 6 h, and then nitrogen adsorption was measured. Finally, nitrogen desorption experiments were carried out at the saturated temperature of liquid nitrogen. The multi-point Brunauer–Emmett–Teller (mBET) theory was used to calculate the surface area, and the average pore diameter was calculated using the Barrett–Joyner–Halenda (BJH) model [22,23].

#### 2.2.6. Determination of Loading Efficiency

Ten mg microscaffolds were accurately weighed, dissolved in 2 mL acetonitrile, and centrifuged at 8000 rpm for 5 min. The supernatant was discarded and replenished with the same amount of acetonitrile. This process was repeated three times and then the residual solvent was volatized. The precipitated protein drug was re-dissolved in 0.8 mL PBS for the BCA assay.

#### 2.2.7. In Vitro Release

Both porous and pore-closed microscaffolds were accurately weighed and dispersed in 1 mL PBS, and then incubated at 37 °C in a shaker at a rate of 100 rpm. The supernatant was collected at a certain interval (1, 2, 4, 7, 10, 14 d), the amount of protein drug was determined and fresh medium was added. After the release period, the residual microscaffolds were freeze-dried and the protein drug content was determined according to the same method as in Section 2.2.6.

#### 2.2.8. Integrity of Protein Drug

Size exclusion chromatography (SEC) was performed to investigate the structural integrity of α-amylase (column: TSK gel 2000 SW; mobile phase: phosphate buffer solution pH 7.4; flow rate: 1 mL/min; wavelength: 214 nm; sample volume: 20 μL).

The detection of the circular dichromatic spectrum (CD spectra) was carried out on the JASCO J-815 circular dichromatic spectrometer (JASCO, Japan Spectral Co. Ltd., Tkoyo, Japan). The natural α-amylase and the prepared pore-closed microparticle were dispersed into water. The detection of circular dichromatic spectrum in the far ultraviolet region of 190~260 nm, data pitch was 0.2 nm, bandwidth was 2 nm, and the scanning speed was 100 nm/min.

#### 2.2.9. Activity of α-Amylase

The activity of α-amylase was determined by the dinitrosalicylic acid solution (DNS) method [24,25]. To prepare the DNS reagent, 10.6 g 3,5-dinitrosalicylic acid was dissolved in 500 mL distilled water in oil bath at 48 °C, then 19.8 g sodium hydroxide solution was added slowly with stirring. A total of 306 g Rochelle salts (sodium potassium tartrate), 7.6 mL phenol (melt at 50 °C), and 8.3 g sodium metabisulfite were added into the solution. When all the components had dissolved, water was added to a total volume of 1416 mL, then it was transferred into an amber bottle and stored for one week before use. A citric buffer (pH 6.0) was prepared with 45.3 g sodium phosphate dodecahydrate and 7.74 g citrate acid with a total volume of 1000 mL.

Different amounts of glucose were dissolved in 0.4 mL distilled water and mixed with 0.8 mL DNS. The mixture was incubated in a boiling water bath for 10 min, and quickly cooled down to room temperature with cold water. Then, the absorbance at 540 nm was obtained to get a standard glucose curve. Similarly, 0.35 mL 1% (*w/v*) soluble starch solution was made with citric buffer, and mixed with 0.05 mL α-amylase solution. The mixture was incubated in an oil bath at 60 °C for 5 min, then 0.8 mL DNS was added quickly to end the reaction and transferred to a boiling water bath for 5 min. The absorbance was read and the amount of hydrolyzed glucose was calculated according to the standard curve.

### 2.3. Statistical Analysis

All experiment tests were repeated five times and data were shown as mean ± standard deviation. *p* < 0.05 was regarded as significant using GraphPad Prism 7 software (GraphPad Prism, Inc., CA, USA). Significant differences were evaluated using two-tailed t test between two groups for in vitro assays.

## 3. Results and Discussion

### 3.1. Investigation on Aqueous Two-Phase Systems and Protein Drugs

Aqueous two-phase systems were investigated to decide what kind of protein drugs have the potential to be partitioned into the porous microscaffolds. A protein drug that tends to distribute in the PEG phase needs to be picked out first. The sulfate and phosphate could lower the absorbance greatly due to their combination with copper ions, the chromogenic agent in the BCA method. In the ATPS, the two phases did not separate completely in fact, but each phase partially contained the other. The uncertainty of salt concentration made it difficult to determine the actual protein drug concentration in the PEG phase, even when the standard curve was corrected with the corresponding salt. Sodium chloride was added to adjust the distribution. The mechanism was that sodium ions and chloride ions had a concentration difference between the two phases, resulting in a potential difference. The partition coefficient *K* of a biomolecule varies exponentially with the electrochemical potential difference between the phases and the net charge of the partitioned biomolecule (Table 1) [26,27,28].

**Table 1:** Partition coefficient (Log K) of different aqueous two-phase systems (ATPSs) and protein drugs. K = protein drug concentration in upper poly(ethylene glycol) (PEG) phase/concentration in lower salt phase.

Formulation	ATPS	NaCl (*w*/*v*)	Protein (1 mg/mL)	Log K
1	PEG4000/potassium phosphate (15%: 10%)	0	α-amylase	−0.45
2	PEG4000/potassium phosphate (15%: 10%)	0.8%	α-amylase	−0.09
3	PEG4000/potassium phosphate (15%: 10%)	1.2%	α-amylase	−0.21
4	PEG4000/ammonium sulfate (15%: 20%)	0	α-amylase	0.30
5	PEG4000/ammonium sulfate (15%: 20%)	0.8%	α-amylase	0.24
6	PEG4000/ammonium sulfate (15%: 20%)	1.2%	α-amylase	0.29
7	PEG4000/ammonium sulfate (15%: 20%)	0	BSA	−0.71
8	PEG4000/ammonium sulfate (15%: 20%)	0.8%	BSA	−0.69
9	PEG4000/ammonium sulfate (15%: 20%)	1.2%	BSA	−0.69

**Figure 1:**
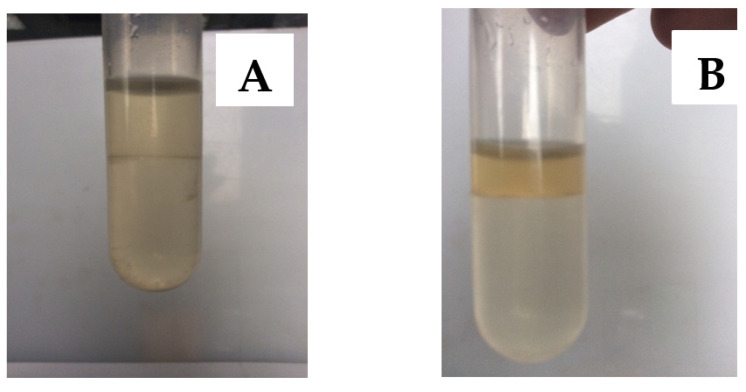
Images of different aqueous two-phase systems with α-amylase. (**A**) Formulation 2, the PEG/phosphate system. (**B**) Formulation 4, the PEG/sulfate system. The upper phase was PEG, the lower phase was salt.

α-amylase was brown in solution, thus can be distinguished directly in the ATPS. Figure 1A shows the PEG/phosphate system (formulation 2) with no significant difference between the upper and lower phases. Figure 1B showed the PEG/sulfate system (formulation 2). It can be clearly seen that the upper PEG phase was darker, indicating a higher α-amylase concentration.

### 3.2. Characterization of Porous Microscaffolds

The porous microscaffolds were prepared by the water-in-oil-in-water (W_1_/O/W_2_) solvent evaporation method, using NH_4_HCO_3_ as a gas-producing agent. The porosity could be adjusted by the proportion of NH_4_HCO_3_, and partly by the PEG because of its leaching process. Neither a too low nor a too high porosity was desired, as a low porosity might limit the protein drug loading capacity and a high porosity might make the pore-closing process difficult [29,30].

**Figure 2:**
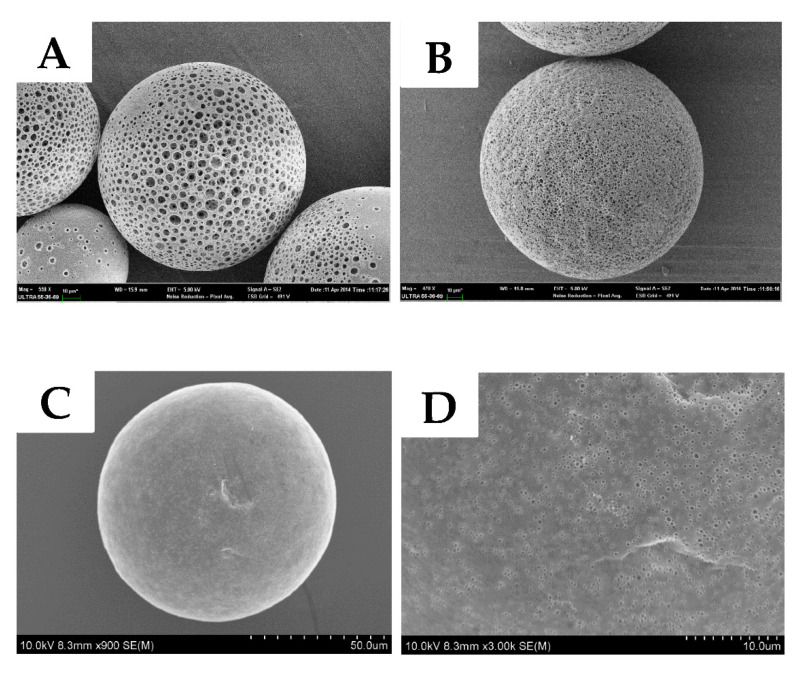

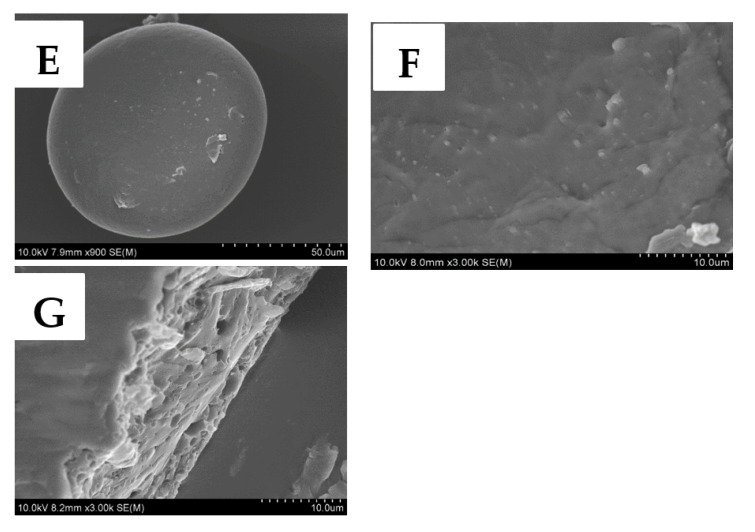
Scanning electron microscope images of different formulations. (**A**) Microscaffolds of 20% NH_4_HCO_3_. (**B**) Microscaffolds of 15% NH_4_HCO_3_. (**C**) Microscaffolds of 10% NH_4_HCO_3_. (**D**) Amplified image of microscaffolds of 10% NH_4_HCO_3_. (**E**) Pore-closed microscaffolds of 10% NH_4_HCO_3_. (**F**) Amplified image of pore-closed microscaffolds of 10% NH_4_HCO_3_. (**G**) Sectional image of pore-closed microscaffolds of 10% NH_4_HCO_3_.

The amount of NH_4_HCO_3_ was differed in formulations of microscaffolds, at a percentage of 10%, 15%, and 20%. According to the SEM images, formulations were roughly selected by the size of the pores and porosity. The porosity and pore size increased directly with the increase amount of NH_4_HCO_3_. Figure 2A,B is the microscaffolds with 20% and 15% NH_4_HCO_3_. Such a highly porous structure might impede the pore-closing process, which was based on the mobility of the polymer. Figure 2C shows the formulation with 10% NH_4_HCO_3_, only small pores were found on the surface due to the lower amount of NH_4_HCO_3_. Figure 2D is an amplified image of Figure 2c. Given that the protein drug was loaded in the solution state, such a pore size was big enough for loading and could facilitate the pore-closing process. Figure 2E,F is the pore-closed microscaffolds heated above the Tg for 24 h. It was clear that the pores were closed successfully and could be used as a sustained depot. Otherwise, the pores would lead to a severe initial burst and a much shorter release period. Figure 2G shows a sectional view of pore-closed microscaffolds. Even though the pores on the surface were closed, the inner porous structure did not disappear completely. Compared with similar studies [21], it was found that the addition of PEG during the microscaffold hardening could inhibit the pore-forming process, resulting in a reduction of porous structure, most probably because PEG enhanced the mobility of polymeric chains of PLGA, and self-encapsulation also happened in the hardening process.

**Figure 3:**
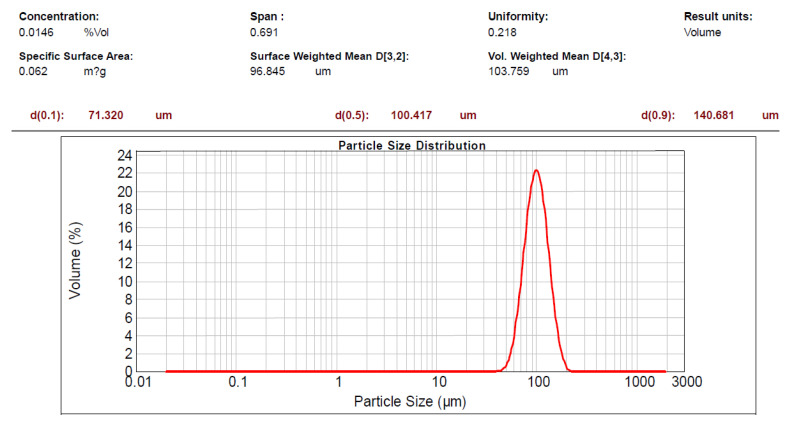
Size distribution of the porous microscaffolds. Formulation contained 10% NH_4_HCO_3_.

Figure 3 shows that the surface weighted average particle size of microscaffolds was 96.84 μm, with a uniformity of 0.218. Hence, it was not necessary to sieve before being used to eliminate the variation of different samples.

**Figure 4:**
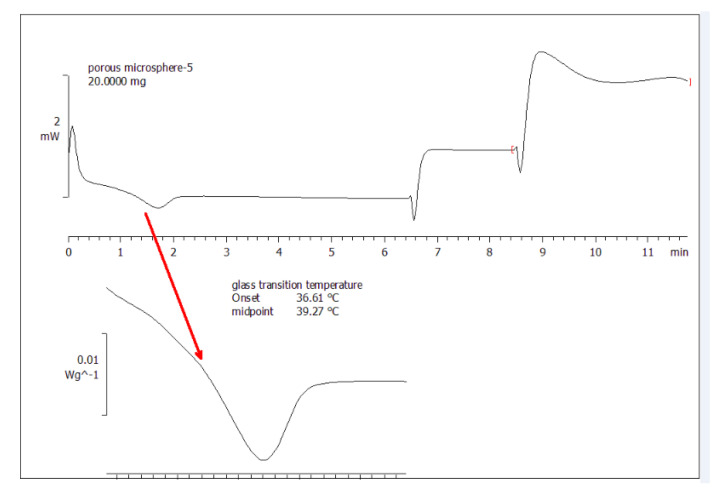
Differential scanning calorimeter thermogram of porous microscaffolds (10% NH_4_HCO_3_). The temperature corresponding to the midpoint of the negative peak was the glass transition temperature.

Figure 4 shows that the glass transition temperature of porous microscaffolds was 39.27 °C. Thus, in the pore-closing process, the temperature was set at 42 °C, slightly higher than the Tg to mobilize the polymeric chains to close the pores. The amount of PEG was found to have an influence on the Tg.

### 3.3. Determination of Loading Amount

According to the mBET theory, the surface areas of blank microscaffolds and pore closed microscaffolds were about 0.998 m^2^ g^−1^ and 1.502 m^2^ g^−1^, respectively. BJH analysis showed that the average pore diameters on blank microscaffolds and pore closed microscaffolds were about 30.578 nm and 3.413 nm, respectively. Therefore, in combination with the results in Figure 2, we know that compared with blank microscaffolds, some pores were closed in microscaffolds loaded with protein and the protein was successfully encapsulated in the microscaffolds. This result was consistent with the findings of Homayun, B. et al. [31].

The key issue of the porous microscaffolds is how to improve the loading amount to emulate the traditional microscaffolds. From Table 2, a high loading amount of 9.67 ± 6.28% could be achieved by employing the aqueous two-phase system compared with the protein drug-only control group (0.16 ± 0.10%). The protein drug loading medium consisted of ammonium sulfate and PEG had a lower loading amount than that of the one of ammonium sulfate-only. This could be attributed to the protein drug being able to stay in the PEG phase out of the microscaffolds rather than get into the pores. On the other hand, formulation 2, which had sulfate only, could force the protein drug to enter the porous structure and stay with the PEG pre-encapsulated in microscaffolds. Formulation 3 with no PEG pre-encapsulated in the microscaffolds showed a much lower loading amount, indicating the necessary presence of PEG.

Furthermore, not only α-amylase can have a high loading amount in PLGA microscaffolds. Any other hydrophobic protein drugs also have the potential to have a high partition coefficient in PEG phase and can be encapsulated in microscaffolds such as lysozyme, thaumatin, and conalbumin [32,33,34]. A PEG–PLGA copolymer has been synthesized and applied already [35,36] and may have better performance because PEG chains were fixed in porous microscaffolds, unlikely to diffuse into the outer sulfate phase.

### 3.4. Release In Vitro

**Figure 5:**
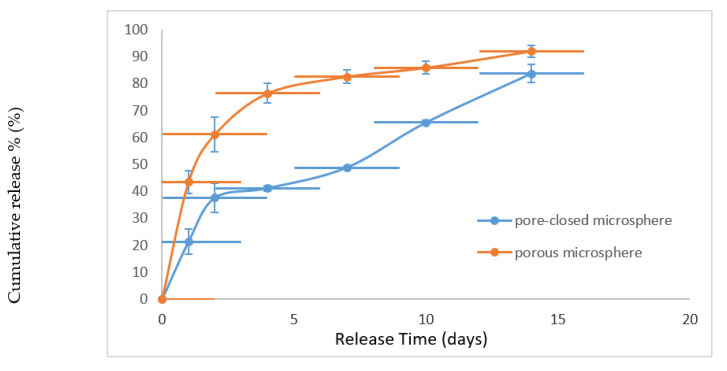
Cumulative release curve of α-amylase. Porous microscaffolds and pore-closed microscaffolds were compared. All data are presented as mean ± SD, n = 3.

Figure 5 shows the cumulative release curve of both pore-closed microscaffolds and the porous microscaffolds. The initial burst of porous particles was severe, with more than 60% released in the first two days, making it unsuitable for sustained release. The unclosed pores became the channels for water, which not only promoted the release of thee protein drug, but also accelerated the degradation of PLGA microscaffolds. The higher cumulative release rate at 14 days could also verify the faster degradation in porous microscaffolds. In contrast, the release profile of pore-closed microscaffolds was more stable. The results were consistent with previous studies of Kang, J. et al. [15]. Generally, PLGA 2A (MW:12000) is used for 1-month sustained release use. Thus, the uncompleted release was normal and the remained protein drug was assayed by dissolving the PLGA with acetonitrile.

### 3.5. Size Exclusion Chromatography

**Figure 6:**
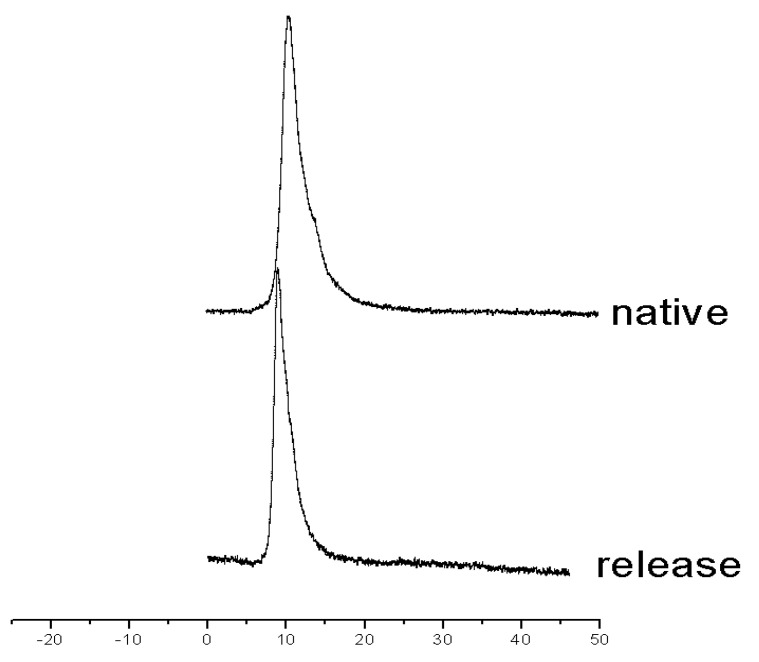
Size exclusion chromatography. The “release” was sample released from pore-closed microscaffolds after one day and the “native” was native α-amylase diluted into a concentration about 0.2 mg/mL.

The chromatography showed a similar retention time of native and released α-amylase. As shown in Figure 6, the native α-amylase had a relatively wide tailing peak, indicating it was a mixture of slightly aggregated α-amylase and monomer. After loading and releasing, the released α-amylase showed a sharp peak, which shared the same retention time of the aggregated part of α-amylase.

**Figure 7:**
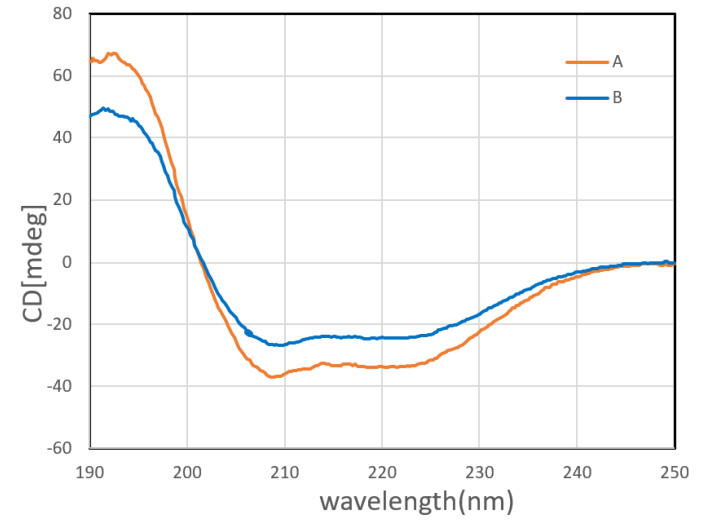
Circular dichroism spectra of (**A**) native α-amylase and (**B**) α-amylase released from pore-closed microscaffolds.

In order to explore whether the structure of the protein encapsulated by the microscaffolds changed during the preparation process, we carried out circular CD spectra on the untreated α-amylase and α-amylase released from pore-closed microscaffolds. Figure 7 showed the far-UV CD spectra of native α-amylase and α-amylase released from pore-closed microscaffolds. The conformational state of released α-amylase was not changed from that of native α-amylase. This indicates that the encapsulation of α-amylase in microscaffolds does not impair its natural conformational state or function. This finding is consistent with previous reports by Paik, D.H. et al., who encapsulated BSA in porous PLGA microspheres, and the encapsulated BSA retained its specific bioactivity [37].

### 3.6. Activity of α-Amylase

The activity was measured by the DNS reagent, based on the reducing sugar produced in its reaction with starch. Relative activity was calculated and the activity of native α-amylase was set as 1. The testing sample was the α-amylase released one day from microscaffolds. The absorbance was converted into the concentration of reducing sugar first, according to the standard curve made by glucose. The amount of protein drug was measured using the BCA method.

**Figure 8:**
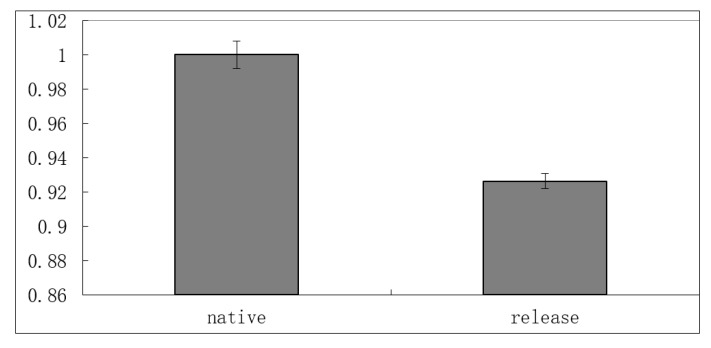
Relative activity of released α-amylase. All data are presented as mean ± SD, n = 3.

From Figure 8, the released α-amylase showed a high relative activity, demonstrating that the preparation method was mild and friendly to α-amylase. Theoretically, the aqueous two-phase system is mild and has been successfully applied to extract and separate protein drugs for decades. Thus, the key factor that may alter the activity of protein drug is the pore-closing process, which is performed at a relatively high temperature (above Tg), usually 30 to 50 °C for PLGA. The α-amylase we used here was a thermostable one, which had an optimum temperature of 60 to 70 °C. Other extremely heat-sensitive protein drugs could be damaged by this pore-closing method. In this case, the pore-closing process needs to be performed at a lower temperature with substances that can facilitate the mobility of polymeric chains, for example, PEG.

## 4. Conclusions

A novel method, encapsulating α-amylase effectively and stably into PLGA microscaffolds was proposed with the application of an aqueous two-phase system. Self-encapsulating microscaffolds increased protein drug loading and promoted the sustained release of encapsulated proteins. The sulfate/PEG system was friendly to protein drugs, avoiding any organic solvent, high temperature, or violent mechanical force used in traditional preparation methods. Protein drugs like α-amylase have the potential to be encapsulated in microscaffolds once it has a high partition coefficient in the PEG/sulfate system. Further efforts are under way to investigate more ATPSs and protein drugs. A PEG–PLGA copolymer may have the potential to simplify this method and have a higher efficiency.

## Figures and Tables

**Table 2:** The loading amount of α-amylase in different formulations. The ammonium sulfate and PEG4000 were the composition of the protein drug loading medium. Microscaffolds were suspended and violently shaken in the medium for 24 h. All data are presented as mean ± SD, n = 3.

Formulation	Ammonium Sulfate (*w*/*v*)	PEG4000 (*w*/*v*)	Microscaffolds	Loading Amount (*w*/*w*)
1	20%	10%	PEG	1.67 ± 0. 23%
2	20%	0	PEG	9.67 ± 6.28%
3	20%	0	NO PEG	1.21 ± 0.52%
4	0	0	PEG	0.16 ± 0.10%

## Data Availability

Not applicable.
